# Study of the Straw Compaction Degree as a Function of Moisture Content, Particle Size and Process Temperature

**DOI:** 10.3390/ma17235869

**Published:** 2024-11-29

**Authors:** Dominik Wilczyński, Krzysztof Talaśka, Krzysztof Wałęsa, Dominik Wojtkowiak, Kuba Kryszczyński, Andrzej Kołodziej, Karol Konecki, Łukasz Urbaniak

**Affiliations:** 1 Faculty of Mechanical Engineering, Institute of Machine Design, Poznan University of Technology, Piotrowo Str. 3, 61-138 Poznań, Poland; krzysztof.talaska@put.poznan.pl (K.T.); krzysztof.walesa@put.poznan.pl (K.W.); dominik.wojtkowiak@put.poznan.pl (D.W.); kuba.kryszczynski@put.poznan.pl (K.K.); 2 Department of Mechanical Engineering, Polytechnic Faculty, University of Kalisz, Poznańska 201-205, 62-800 Kalisz, Poland; a.kolodziej@uniwersytetkaliski.edu.pl (A.K.); k.konecki@uniwersytetkaliski.edu.pl (K.K.); l.urbaniak@uniwersytetkaliski.edu.pl (Ł.U.)

**Keywords:** straw, triticale, compaction, compaction degree, solid fuel, biomass, ANOVA analysis

## Abstract

The paper presents research on the process of densifying rye-wheat straw for its use in producing mouldable biofuel. The straw used in the research is a waste material from a farm located in Wielkopolska, resulting from the cultivation of triticale for the purpose of producing feed for pig farming. The aim of the study is to determine the utilisation of this material for the production of an agglomerate for energy purposes, such as heating the farm’s infrastructure. The research was conducted for two moisture levels of straw: *M* = 10% and 30%. Before the experiment, the straw was cut into particles of the desired size: *S* = 10, 20, 30, 40, 50 and 60 mm. The densification process was carried out at temperatures *T* = 25, 50, 100, 150 and 200 °C, subjecting the straw to a compaction pressure of 15 MPa. Based on experimental studies, two values of the densification degree were determined: *x*_1_—the densification degree under load; and *x*_2_—the densification degree after unloading. The densification degree *x*_2_ is more relevant from the perspective of storage and transport. ANOVA analysis of the results showed that the most significant factors affecting *x*_1_ were particle size S and process temperature T, with higher *x*_1_ values obtained for straw moisture of 30%. The ANOVA analysis of the densification degree after unloading (*x*_2_) revealed that higher x_2_ values were achieved for straw with 10% moisture and the smallest particle size of 10 mm. The most significant factors affecting *x*_2_ were particle size and moisture content. Studies of the friction coefficient between the straw and the materials of the densification equipment components indicated that the optimal process temperature is 150 °C. The conducted research and the obtained results determined the optimal input parameters for the process and also provided a solid support for further studies, including investigation of the influence of other factors, such as binders.

## 1. Introduction

Research on the biomass densification process is very popular and widely discussed by scientists from around the world, due to the production and subsequent use of biofuels in the form of briquettes or pellets. Among the most important sources of biofuels are agricultural residues, such as straw [1]. Its share in biofuel production, alongside woody biomass, is considered one of the most significant in Central Europe [2,3]. The low density of biomass is approximately 80–100 kg/m³ for straw, while for woody biomass it ranges from 150–200 kg/m^3^ [4,5,6]. Other sources report an average density of straw and grasses at around 100–200 kg/m^3^ [7]. Biofuel obtained through the densification process is characterised by low moisture content and allows for a high energy yield [8].

Scientists are studying the biomass densification process, specifically the impact of various factors on this process. These factors mainly include the densification pressure, the moisture content of the material being densified, the type of material being densified, the process temperature, the degree of particle-size reduction, the use of binder additives and the mixing of raw materials in different proportions [4,8,9,10,11,12,13]. The content of lignin, cellulose and hemicellulose also significantly affects the properties of the resulting agglomerate [14,15,16,17,18].

Various types of equipment are used to carry out the densification process. These include granulators, briquette machines, screw extruders, hydraulic piston presses, roller presses, tablet machines and agglomerators. Such equipment is used worldwide to produce biofuels with a uniform structure and the required physical properties [4,19,20,21].

Patta Granado et al. conducted a study on the densification of cassava using a single-piston press. The material was densified under three different pressure values: 102, 153 and 204 MPa, with different exposure times of 0, 60 and 120 s. The material was not densified at elevated temperatures. For the densification pressure of 204 MPa and a duration of 120 s, the highest density agglomerate was obtained, which also exhibited the highest strength and stability in terms of form and shape. The authors concluded that densifying this biomass material is a good solution to the problems of transporting and storing undensified cassava [22].

Höntsch et al. carried out research on the densification process of wheat straw. Among other things, they examined the influence of the degree of particle size reduction achieved by different techniques on the energy efficiency of the process and the mechanical properties of the briquette. The authors found that the densification pressure should not be too high, as part of the energy is lost to elastic compression of the material, which does not improve the quality of the briquette. Densifying the straw material in its fibrous or highly fragmented form positively influenced the mechanical properties of the agglomerate [23].

Stelte et al. conducted studies on the pelletisation of materials, including beech, spruce and straw. The study focused on determining the strength and integrity of the pellets as a function of the input parameters of the densification process. The results showed an increase in the compressive strength of the pellets produced at higher temperatures. Densification at higher temperatures caused cracks, likely due to the phenomenon of lignin distribution during the process. This effect was observed only in pellets made from spruce and beech, and did not apply to the pellets made from straw. Overall, the studies demonstrated that both temperature and chemical composition have a significant impact on the quality and cohesion of the resulting biofuel [24].

Adapa et al. conducted studies on the densification process of barley straw, rapeseed, oat, and wheat straw. These materials were densified using four different pressure levels. It was observed that a pressure of 63.2 MPa for barley and wheat, and a pressure of 94.7 MPa for rapeseed and oat, allowed for the production of an agglomerate with the highest density [17]. Matkowski et al. conducted studies on the densification of pure straw and straw mixed with additives such as calcium carbonate or cassava starch. The densification was carried out for materials with a moisture content of 20% at a temperature of 78 °C in a sleeve with a height of 80 mm, adding additives in amounts of 50, 100 and 150 mg. The mixture of straw with 6% calcium carbonate showed the most uniform particle-size distribution and the lowest geometric mean particle size of 0.71 mm, which had a positive effect on the formation of bonds between particles, the densification process and the quality of the agglomerate. The resulting pellets exhibited a higher elastic modulus and high compressive strength [25].

Bai et al. conducted studies on the densification process of torrefied wheat straw and peanut shells with the addition of water. The results showed that optimal process conditions were achieved with 15% peanut shell content and 10% water content. The bulk density and maximum strength of the agglomerate initially decreased and then increased. Energy consumption rose with increasing temperature. The peanut shells, the water content and the friction properties of the biochar were the main factors affecting the cohesion of the pellet mixture. The authors concluded that peanut shells are an effective and inexpensive binder for producing high-quality pellets [26].

Lisowski et al. conducted studies on the physicochemical properties of agglomerates made from hay, wheat straw and their 50/50% mixture. The researchers found that the densification process led to a reduction in the materials’ moisture content. The authors determined the durability index, which showed a negative correlation with both the particle size of the densified material and its moisture content [27].

Adam et al. studied the densification process of a mixture of pine and spruce sawdust using an industrial piston machine from ATNA Industrial Solutions GmbH (Leipzig, Germany) type GreenLine S50. The material was densified under pressures ranging from 100 to 200 MPa, with an initial densification pressure of 13 to 40 MPa. The authors concluded that the duration of pressure exposure had the greatest impact on biomass densification, and the density distribution in the agglomerate varied due to friction occurring on the walls of the forming sleeve. This phenomenon could potentially be improved by using convergent geometry for the inner walls of the forming sleeve [28].

Chen et al., along with his team, conducted studies on the densification of rice straw using a ring-type briquetting machine 9JY-2000A, Shijiazhuang City, China. The main factors influencing the friction coefficient were the density of the briquette, moisture content of the biomass and compression speed. The authors focused on the friction of the material against the forming channels, as they also assessed the wear of the machine’s working components using a scanning electron microscopewith tungsten filament lamp (SU3500, Shanghai, China) [29].

Based on the above review, the research presented in this paper was conducted on the densification process of rye-wheat straw under conditions of low energy consumption, considering a compressive stress of 15 MPa. Additionally, the decision was made to investigate the effect of moisture content, particle size and temperature on the densification degree. Referring to the cited publications on straw densification, the authors wanted the presented study to focus in detail on the impact of particle-size reduction, moisture content and temperature as the main factors influencing the densification degree, which, in turn, is a crucial response of the process. Additionally, the authors used a stamp-sleeve densifying set with a diameter of 20 mm. By introducing process input variables in the experimental studies, such as particle sizes ranging from 10–60 mm with 10 mm increments, process temperatures of 25, 50, 100, 150 and 200 °C and two levels of straw moisture content (10% [25] and 30%), the authors aimed to determine the characteristics of the changes in the process response, in the form of densification degree, as a function of the aforementioned input parameters. Experimental tests were carried out on custom-built research set-ups, including the determination of the friction coefficient. The obtained results were analysed using ANOVA (Analysis of Variance), which, in conjunction with the friction coefficient studies, allowed for the determination of the optimal process settings for densifying straw while achieving agglomerates with good physical and mechanical properties. The densification process was carried out using the testing machine MTS Insight 50 kN (Eden Prairie, MN, USA).

## 2. Materials and Methods

### Material Preparation

The material used in the study was rye-wheat straw (Triticosecale), which was collected as post-harvest biomass from agricultural fields located in the Greater Poland region (west-central Poland) at the geographical coordinates 52°00′08.0″ N, 17°46′46.9″ E. The straw was seasoned for a period of three months after harvest in a closed room at room temperature.

**Figure 1:**
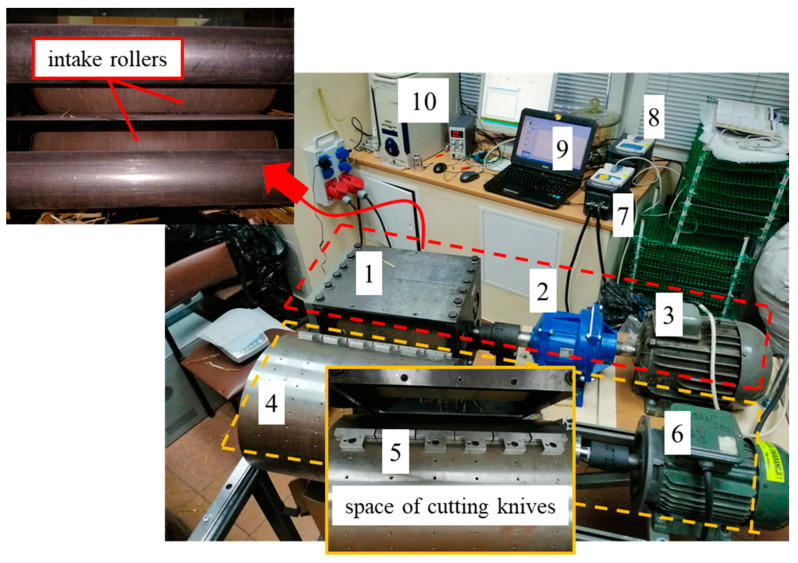
View of the stand used for cutting straw before compaction, into particles of specified lengths. 1—pull-in rollers, 2—reduction gear transmission (Motovario, model HA62, Alpharetta, GA, USA), 3—electric motors of the feeding assembly, 4—rotary drum, 5—cutting knives, 6—electric motor of the cutting assembly, 7—inverter (Schneider Electric, model MX pro 4V2.2 MP4U22AAB, Rueil-Malmaison, France), 8—inverter (Schneider Electric, model MX pro 4V4.0 MP4U40AAB), 9—computer, 10—computer.

**Figure 2:**
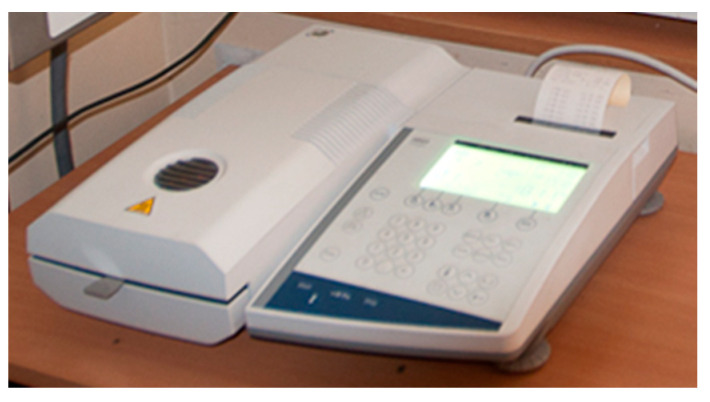
Measuring the moisture content with the Mettler Toledo analyser.

Two levels of moisture content were used in the study. The first moisture level was approximately 10%, and it was classified as dry straw. The second moisture level was 30%, achieved by spraying the straw with water and stabilising the moisture content to the desired value in closed containers. This straw is referred to as wet straw. Before humidifying, the straw was cut into particle sizes of 10, 20, 30, 40, 50 and 60 mm. The same procedure was applied to the dry straw. The equipment used for this process is shown in Figure 1. The moisture content of the straw was measured using a Mettler Toledo moisture analyser (Mettler Toledo, type HR83; Mettler-Toledo AG, 2009, Urdorf, Switzerland) (see Figure 2).

The set-up described above consists of a module with feed rollers (red frame) and a cutting knife module (yellow frame). Both modules are powered by separate electric motors 3 and 6. Motor 3 drives the feed rollers through a reduction gearbox 2 to increase the torque of the feed rollers 1. The cutting knife module (yellow frame) is directly driven by motor 6. Between the cutting drum 4 and motor 6, a torque sensor 5 is installed. Electric motors 3 and 6 are powered via inverters 7 and 8, which are controlled by software installed on computers 9 and 10 Software named MatriX 3.2 build 3397 dedicated for inverters Schneider Electric Company.

**Figure 3:**
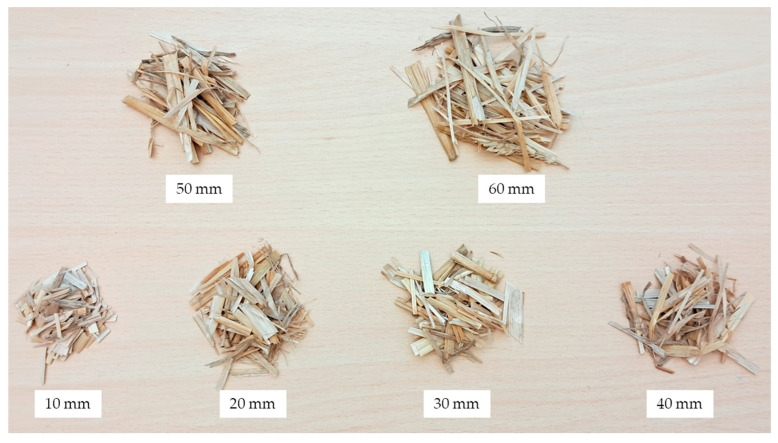
View of the straw samples cut to the required lengths.

The required rotational speed of the feed rollers was set to achieve the desired linear speed of the fed straw, while also transporting it to the cutting knives (Figure 2), where the straw was cut. Cutting the straw into specific lengths also required setting the appropriate rotational speed of the cutting drum 4. This allowed for the production of straw segments with the desired lengths, which were later used in the densification studies. Figure 3 shows the straw samples with the lengths used in the research.

**Figure 4:**
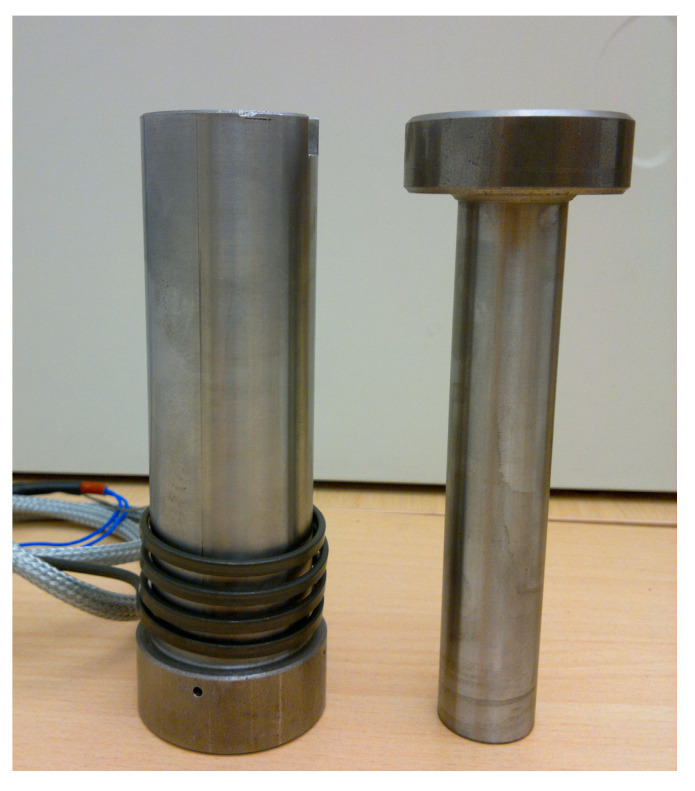
View of the piston-sleeve assembly used in the experiments.

**Figure 5:**
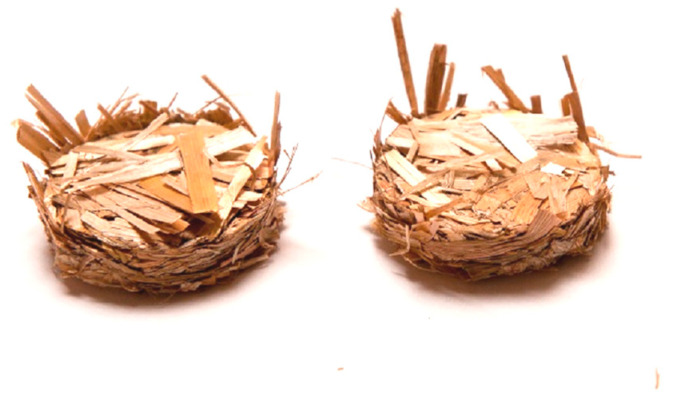
A view of example samples obtained after compacting straw with a particle size of *S* = 50 mm, at a temperature of *T* = 50 °C and moisture content *M* = 10%.

The chopped straw was placed in a sleeve with an inner diameter of 20 mm (in accordance with the standard DIN 51731) [30] (Figure 4), after which a preliminary densification was performed by placing a 50 g weight on the densifying stamp. Subsequently, the straw was densified using a force that allowed achieving a compressive stress of 15 MPa (see Figure 5) by using the strength testing machine MTS Insight 50 kN. This value was chosen in order to study the densification degree of rye-wheat straw while maintaining low energy consumption during the process. For each group of process parameters, such as particle size, moisture content and temperature, the test was repeated 20 times.

The obtained results of the experimental studies were subjected to a multifactorial Analysis of Variance (ANOVA) using the Response Surface statistical model to determine the correlation between the parameters (settings) of the densification process, such as particle size (*S*), process temperature (*T*) and moisture content (*M*), and the response in the form of the densification degree. The parameters used in the ANOVA analysis are summarised in the Table 1, Section 3. The values of the degree of compaction under load *x*_1_ and after unloading *x*_2_ were determined based on the relations (1) and (2), see below.

For each straw length, the densification process was repeated 20 times. For each trial, the densification degree was calculated according to the following formula:

(1) $$x_{1}=\frac{h_{bc}}{h_{acul}}$$

(2) $$x_{1}=\frac{h_{bc}}{h_{acul}}$$

where:

*x*_1_—densification degree under the load applied by the strength testing machine, maintaining a compressive stress of 15 MPa,

*x*_2_—densification degree under unloading of the densified sample, i.e., when the compressive stress is no longer applied,

*h_bc_*—height of the sample before densification mm,

*h_acul_*—height of the sample after densification under the applied compressive stress of 15 MPa mm,

*h_acau_*—height of the sample after densification without the applied compressive stress of 15 MPa mm.

## 3. Results and Discussion

In Table 1, the average values of the densification degree for all the conditions applied during the straw densification process are presented. It should be remembered that for each process setting value the test was repeated 20 times.

The above research results were used for an Analysis of Variance (ANOVA). Table 2 presents the input data and the responses used for the analysis.

### 3.1. Multivariate Analysis of Compaction Degree Under Load x_1_

**Figure 6:**
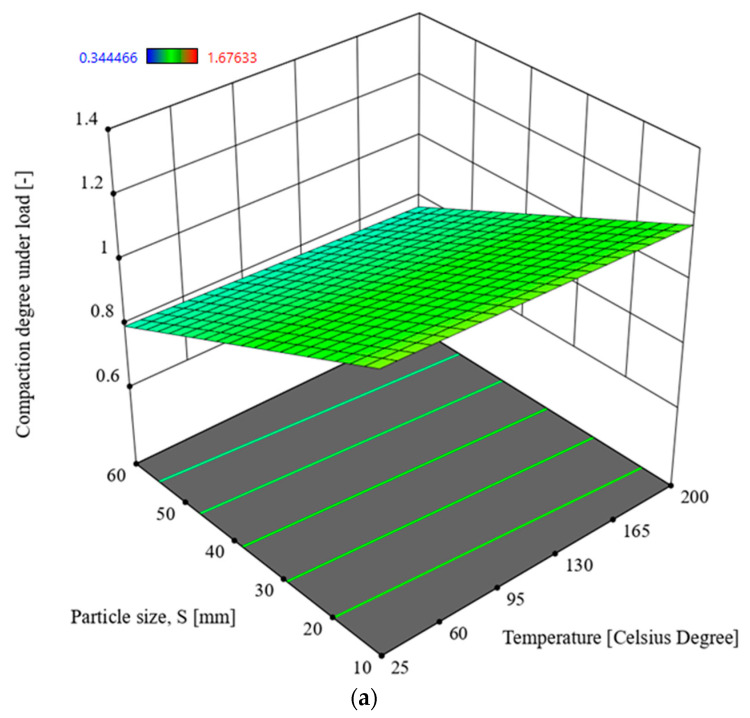

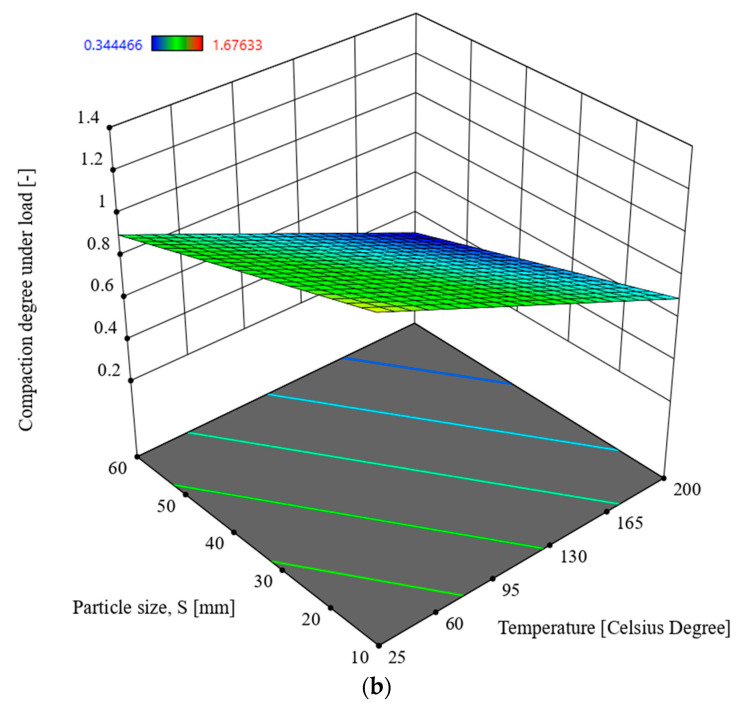
Compaction degree *x_2_* in the function of the particle size *S* and temperature *T* for moisture of straw: (**a**) 10%, (**b**) 30%.

In order to determine the interactions between the input parameters of the process and the response in the form of the compaction degree under the load *x*_1_, Analysis of Variance (ANOVA) was used. The analysis utilised the Reduced 2FI model, which is significant for the analysed parameters and response. This model is characterised by a significance level of *p* = 0.1 and an R^2^ = 0.6225. The model’s F-statistic value is 22.68 (see Table 3), indicating that the model is significant. The individual components of the model have a significance level p lower than 0.05, which makes the model significant. By using the model described by the relationship (3), it is possible to navigate the experimental design space, as the condition is met: (Predicted R^2^—Adjusted R^2^) < 0.2 and Adeg Precision = 32.444. The model graph is shown in Figure 6.

*x*_1_ = 1.09292 − 0.008157 × *S* + 0.003165 × *T* + 0.013259 × *M* − 0.000223 × *T* × *M*, (3)

*x*_1_—compaction degree under load [-], *S*—particle size [mm], *T*—temperature [°C], *M*—moisture [%].

Based on the analysis of the parameter values from Table 3, the value of the particle size *S* (F = 34.29) has a significant influence on the increase in compaction degree *x*_1_. The highest densification degree was obtained for a particle size of *S* = 10 mm, a temperature of *T* = 25 °C and a straw moisture content of *M* = 30%.

### 3.2. Multivariate Analysis of Compaction Degree After Unloading x_2_

**Figure 7:**
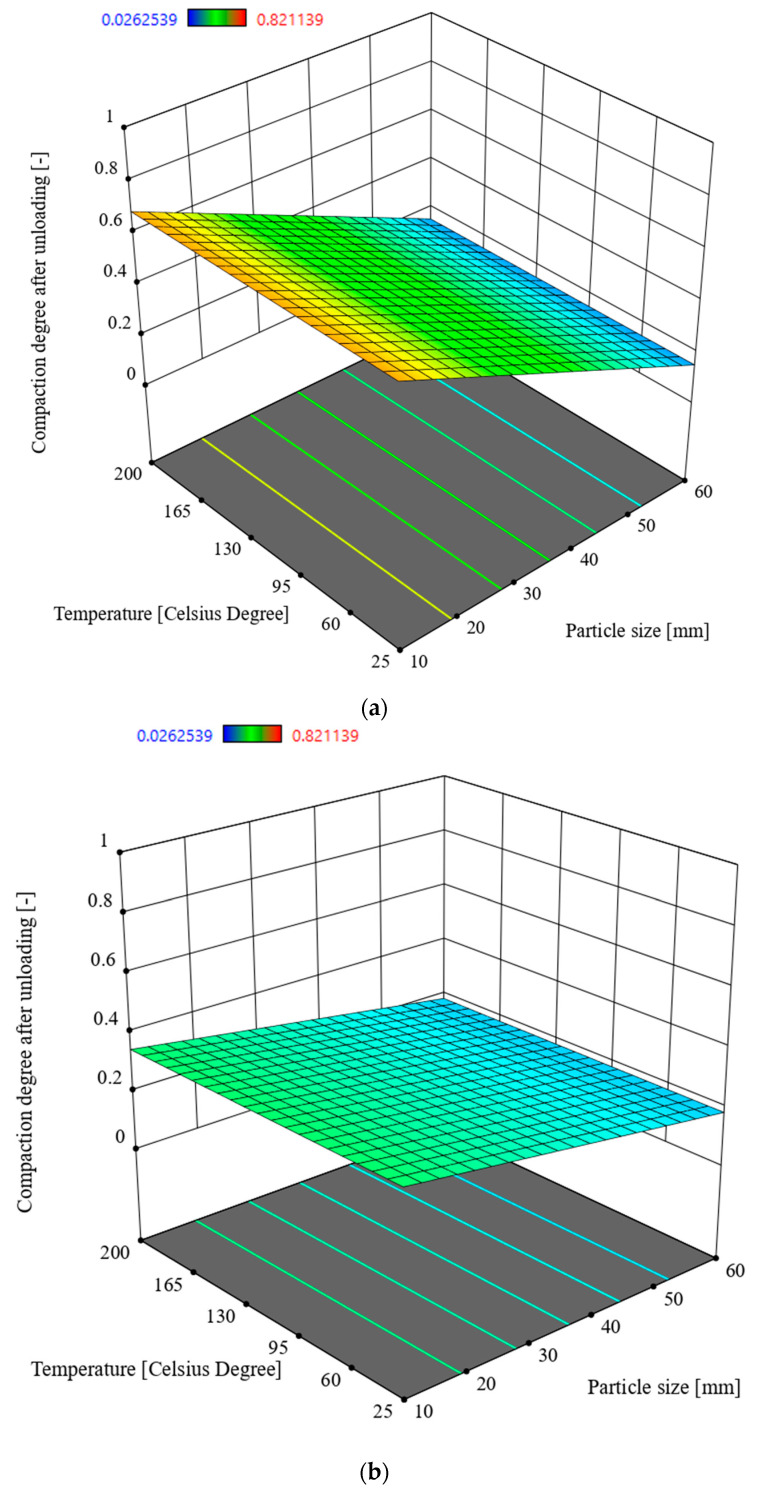
Compaction degree *x_2_* in the function of the particle size *S* and temperature *T* for moisture of straw: (**a**) 10%, (**b**) 30%.

In order to determine the interactions between the input parameters of the process and the response in the form of the compaction degree under the load *x*_2_, Analysis of Variance (ANOVA) was used. The analysis utilised the Reduced 2FI model, which is significant for the analysed parameters and response. This model is characterised by a significance level of *p* = 0.1 and an R^2^ = 0.5779. The model’s F-statistic value is 25.56 (see Table 4), indicating that the model is significant. The individual components of the model have a significance level p lower than 0.05, which makes the model significant. By using the model described by the relationship (4), it is possible to navigate the experimental design space, as the condition is met: (Predicted R^2^—Adjusted R^2^) < 0.2 and Adeg Precision = 15.0163. The model graph is shown in Figure 7.

*x*_2_ = 1.21342 − 0.018165 × *S* − 0.027808 × *M +* 0.000497 × *S* × *M*, (4)

*x*_2_—compaction degree after unloading [-], *S*—particle size [mm], *M*—moisture [%].

Based on analysis of the data from Table 4. Analysing, the particle size *S* has the greatest influence on the densification degree *x_2_*, with a statistic value of F = 44.74. Following this, in hierarchical order, the moisture content of the straw has an impact (F = 19.19) followed by the interaction between two factors, namely particle size *S* and moisture content *M* (F = 12.74). According to the characteristics shown in Figure 7, the highest densification degree is achieved at a lower moisture content *M* = 10% and the smallest particle size. In accordance with Equation (4) and Table 4, temperature does not have a significant influence on the densification degree *x_2_*.

### 3.3. Selection of Densification Process Parameters for Straw Using Recorded Experimental Results

Based on the previous experimental results, further studies were conducted to determine the friction coefficient *μ* of the straw as a function of temperature for densified particles with a size of *S* = 10 mm and moisture content of *M* = 10%, as these parameters yielded the highest densification degree after unloading *x*_2_. This densification degree was considered more significant than the densification under load *x*_1_, as it reflects the storage condition of the briquette, i.e., without applied external pressure.

**Figure 8:**
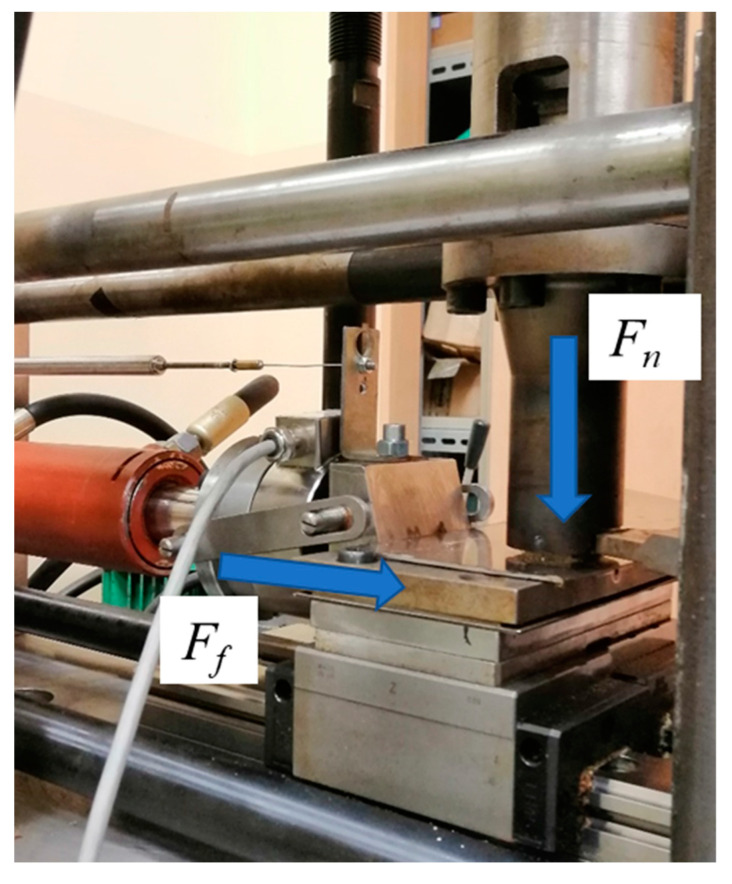
View of the test stand for determining the coefficient of friction as a function of compaction pressure and temperature.

The tests stand which was used for these tests is shown in Figure 8 (general view).

**Figure 9:**
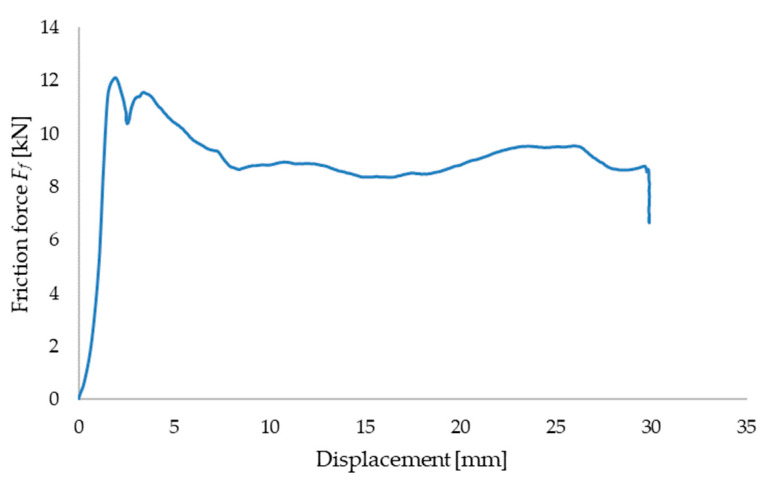
An example of the characteristic curve showing the variation of the friction force as a function of displacement for a densification temperature of *T* = 25 °C and moisture content *M* = 10%.

The rye-wheat straw with parameters *S* = 10 mm and *M* = 10% was densified under a pressure of 15 MPa by applying an appropriate force *F_n_* in the densification sleeve. Afterwards, a steel plate was moved relative to the densified sample and the friction force *F_f_* was measured using an HBM force sensor (HBK Hottinger Brüel and Kjaer Company, Darmstadt, Germany). Figure 9 illustrates an example of the variation in the friction force *F_f_* as a function of the displacement of the piston in the actuator, which moves the steel plate relative to the densified straw sample.

**Figure 10:**
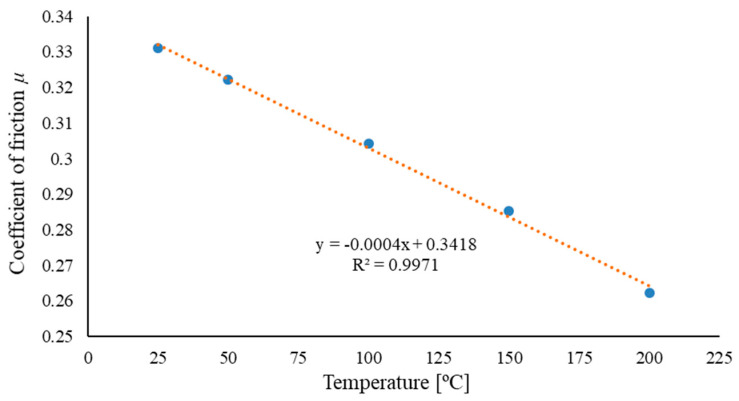
The values of the friction coefficient for the different densification temperatures of straw with parameters *S* = 10 mm, *M* = 10%.

Figure 10 shows the average values of the friction coefficient as a function of temperature. As the temperature increases, there is a slight decrease in the friction coefficient from *μ* = 0.33 at *T* = 25 °C to approximately *μ* = 0.26 at *T* = 200 °C.

Table 5 presents a hierarchical arrangement of the factors and their relationships based on the statistical model values (F-statistics). The table includes the following parameters: *S*—particle size [mm], *T*—temperature [°C], *M*—moisture content [%]. These factors are analysed in relation to the responses being the densification degree under load *x*_1_ [-] and the densification degree after unloading *x*_2_ [-], which represent the densification characteristics observed in the experiment.

Based on the obtained characteristics from the multi-criteria ANOVA analysis, it was found that a higher densification degree *x*_1_ was achieved for lower moisture content of the straw *M* = 10%. The smaller particle size also contributed to the higher *x_1_* value. A smaller particle size also contributed to a higher *x*_1_ value. The process had a negative impact on the straw densification at a moisture content of 30% as an increase in temperature for this moisture level resulted in a decrease in densification degree *x*_1_. For straw with a moisture content of 10%, temperature did not have a significant effect on densification. When considering the densification degree after unloading *x*_2_, the highest densification was achieved for straw with a moisture content of 10% and the smallest particle size *S*. Tumuluru (2019) [31] also demonstrated that briquettes produced from biomass ground using a 12.7 mm screen exhibited higher strength compared to those produced with a 4.8 mm screen. As shown in Table 5 particle size *S* and moisture content *M* had the most significant influence on the densification process.

The lignin content in the densified biomass material influenced the elastic properties of the agglomerate [32,33,34,35,36]. High relaxation of the agglomerate after unloading, referred to as decompression, leads to the degradation of the bonds formed between particles during densification. This means that while the presence of lignin is beneficial in some cases, it can also negatively affect the mechanical properties of the agglomerate, particularly in terms of transport and storage [37,38,39]. Looking at the characteristic changes in the friction coefficient (see Figure 10), specifically the decrease in the coefficient with increasing densification temperature, it can be concluded that the densification process for straw should ideally be conducted at 200 °C. However, examining the characteristics presented in Figure 6 and Figure 7, it is evident that too high a temperature results in a reduced densification degree. This implies that at higher temperatures the straw’s elasticity increases, which directly impacts the densification process. This relationship has been highlighted in previous research [40]. Nalladurai et al. [38,39,40,41] also address this issue, stating that lignin, when exposed to compressive pressure, moisture, and a temperature of around 120 °C, becomes plasticised. This means that during biomass densification, the elastic forces of lignin must be overcome to reach its plasticised state, enabling permanent deformation of the material particles and the formation of new bonds between the particles. Therefore, considering the input parameters for the densification process, it is recommended to limit the process temperature to 150 °C, even at the cost of a slightly higher friction coefficient (see Figure 10). At this temperature, lignin is plasticised and spreads well across other biomass particles, binding cellulose and hemicellulose. Densifying at higher temperatures is unnecessary and inefficient, as achieving higher process temperatures requires additional energy input. This perspective is also supported by Sitzmann and Buschhart [42].

## 4. Conclusions

The conducted research on the densification process of waste material in the form of triticale straw, with moisture content levels of 10% and 30%, densification pressure of 15 MPa, particle sizes ranging from 10–60 mm with 10 mm increments and process temperatures of 25 °C, 50 °C, 100 °C, 150 °C and 200 °C as process settings enabled the determination of responses in the form of densification degree values, *x*_1_ and *x*_2_. Using Analysis of Variance (ANOVA) between the mentioned settings (input parameters) and the process responses, while also complementing the study with an experiment to determine the friction coefficient of the straw material, the following conclusions were drawn.

1. Based on the experimental studies, two values of densification degree were determined: *x*_1_, the densification degree under load, and *x*_2_, the densification degree after unloading. The densification degree *x*_2_ is considered more significant in terms of storage and transport. This is because, for materials like biomass, the ability of the densified material to maintain its shape and structural integrity after unloading is crucial for practical applications such as transportation and storage. A higher *x*_2_ value indicates better stability of the agglomerate after compression, which is desirable for ease of handling and minimising the risk of re-expansion or degradation during storage and transport.
2. The Analysis of Variance (ANOVA) of the obtained results showed that the particle size *S* and the process temperature *T* had the greatest influence on the value of *x*_1_ (the densification degree under load). Higher values of *x*_1_ were achieved for straw with a moisture content of 30% (see Figure 6 and Table 3 and Table 5).
3. The Analysis of Variance (ANOVA) regarding the densification degree after unloading *x*_2_ showed that higher values of *x_2_* were obtained for straw with a moisture content of 10% and the smallest particle size of *S* = 10 mm. The greatest influence on the *x_2_* value was exerted by the particle size and moisture content (see Table 5 and Figure 7).
4. The conducted studies on the coefficient of friction of straw against the components of the densifying device indicated that the optimal process temperature is *T* = 150 °C. This temperature allows for maintaining lower energy consumption in the process, even though the friction coefficient of the straw increases at this temperature. The lowest value of the friction coefficient was recorded at a densification temperature of 200 °C.
5. To extend the conducted research and obtain more detailed information, the authors believe that the study could be expanded by investigating the densification of straw at moisture content levels lower than 10%. Additionally, the influence of compaction stress could be explored by densifying straw at stress levels lower than 15 MPa, such as 10 MPa, and higher stress levels, for example, 20, 25 MPa, or even higher. Furthermore, to achieve lower energy consumption in the process while maintaining good mechanical properties of the resulting agglomerate, different types of binders could be used in the study to determine their impact on the mechanical properties of the agglomerate.

## Figures and Tables

**Table 1:** The averaged values of the experimental results from 20 trials are presented as responses to the specific input settings of the process.

Particle Size *S* [mm]	Temperature *T* [°C]	Moisture *M* [%]	Compaction Degree Under Load *x*_1_ [-]	Compaction Degree After Unloading *x*_2_ [-]
10	25	10	1.12	0.82
10	50	10	1.16	0.67
10	100	10	1.15	0.61
10	150	10	1.39	0.74
10	200	10	1.19	0.76
20	25	10	0.98	0.48
20	50	10	1.03	0.49
20	100	10	1.15	0.52
20	150	10	1.02	0.43
20	200	10	1.09	0.62
30	25	10	1.14	0.59
30	50	10	1.06	0.59
30	100	10	1.05	0.44
30	150	10	0.99	0.38
30	200	10	1.14	0.60
40	25	10	1.19	0.63
40	50	10	0.99	0.44
40	100	10	0.78	0.25
40	150	10	0.87	0.26
40	200	10	0.91	0.32
50	25	10	0.95	0.36
50	50	10	0.78	0.27
50	100	10	0.88	0.21
50	150	10	0.76	0.08
50	200	10	0.81	0.19
60	25	10	0.84	0.29
60	50	10	0.82	0.29
60	100	10	0.77	0.15
60	150	10	0.82	0.05
60	200	10	0.78	0.09
10	25	30	1.68	0.66
10	50	30	1.13	0.19
10	100	30	0.52	0.26
10	150	30	0.79	0.02
10	200	30	1.24	0.69
20	25	30	1.49	0.41
20	50	30	0.99	0.42
20	100	30	0.48	0.34
20	150	30	0.68	0.15
20	200	30	0.78	0.13
30	25	30	1.58	0.61
30	50	30	0.99	0.17
30	100	30	0.60	0.23
30	150	30	0.54	0.18
30	200	30	0.95	0.19
40	25	30	1.29	0.31
40	50	30	1.02	0.03
40	100	30	0.76	0.21
40	150	30	0.52	0.34
40	200	30	0.53	0.22
50	25	30	1.13	0.22
50	50	30	0.92	0.05
50	100	30	0.62	0.25
50	150	30	0.49	0.35
50	200	30	0.40	0.38
60	25	30	0.97	0.15
60	50	30	0.79	0.04
60	100	30	0.54	0.18
60	150	30	0.42	0.24
60	200	30	0.34	0.26

**Table 2:** A summary of the input coefficients and the responses used in the conducted ANOVA analysis.

Name	Units	Type	Low	High
Particle size, *S*	[mm]	Factor	10	60
Temperature, *T*	[°C]	Factor	25	200
Moisture, *M*	[%]	Factor	10	30
Compaction degree under load, *x*_1_	[-]	Response	0.34	1.68
Compaction degree after unloading, *x*_2_	[-]	Response	0.03	0.82

**Table 3:** ANOVA results. Dependent variable—compaction degree under load—*x*_1_ [-].

Source	Sum of Squares	df ^a^	Mean Square	F-Value	*p*-Value	
Model	3.08	4	0.7701	22.68	<0.0001	significant
*S*	1.16	1	1.16	34.29	<0.0001	
*T*	0.8549	1	0.8549	25.17	<0.0001	
*M*	0.4625	1	0.4625	13.62	0.0005	
*T · M*	0.6998	1	0.6998	20.61	<0.0001	

^a^ degrees of freedom.

**Table 4:** ANOVA results. Dependent variable—compaction degree after unloading—*x*_2_ [-].

Source	Sum of Squares	df ^a^	Mean Square	F-Value	*p*-Value	
Model	1.46	3	0.4876	25.56	<0.0001	significant
*S*	0.8536	1	0.8536	44.74	<0.0001	
*M*	0.3661	1	0.3661	19.19	<0.0001	
*S **^.^** M*	0.2431	1	0.2431	12.74	0.0007	

^a^ degrees of freedom.

**Table 5:** Hierarchy of components of individual models based on the F-value criterion.

Response	1	2	3	4	5
*x*_1_	*S*	*T*	*T · M*	*M*	-
*x*_2_	*S*	*M*	*S · M*	-	-

## Data Availability

The original contributions presented in this study are included in the article. Further inquiries can be directed to the corresponding author.
